# Preparation and Rheological Evaluation of Thiol–Maleimide/Thiol–Thiol Double Self-Crosslinking Hyaluronic Acid-Based Hydrogels as Dermal Fillers for Aesthetic Medicine

**DOI:** 10.3390/gels10120776

**Published:** 2024-11-28

**Authors:** Chia-Wei Chu, Wei-Jie Cheng, Bang-Yu Wen, Yu-Kai Liang, Ming-Thau Sheu, Ling-Chun Chen, Hong-Liang Lin

**Affiliations:** 1 School of Pharmacy, College of Pharmacy, Kaohsiung Medical University, Kaohsiung 807378, Taiwan; eric.vaccine@gmail.com; 2 School of Pharmacy, College of Pharmacy, Taipei Medical University, Taipei 11031, Taiwan; englave01@tmu.edu.tw (W.-J.C.); mingsheu@tmu.edu.tw (M.-T.S.); 3 TMU Research Center of Drug Discovery, Taipei Medical University, Taipei 11031, Taiwan; 4 Department of Biotechnology and Pharmaceutical Technology, Yuanpei University of Medical Technology, Hsinchu 30015, Taiwan; opu98781@gmail.com (B.-Y.W.); d8801004@mail.ypu.edu.tw (L.-C.C.); 5 Department of Pharmacy, Chia-Nan University of Pharmacy and Science, Tainan 71710, Taiwan; apccac1004@gmail.com

**Keywords:** double self-crosslinked hydrogels (*dsc*HA), hyaluronic acid (HA), thiol–maleimide click chemistry, dermal fillers, facial rejuvenation

## Abstract

This study presents the development of thiol–maleimide/thiol–thiol double self-crosslinking hyaluronic acid-based (*dsc*HA) hydrogels for use as dermal fillers. Hyaluronic acid with varying degrees of maleimide substitution (10%, 20%, and 30%) was synthesized and characterized, and *dsc*HA hydrogels were fabricated using two molecular weights of four-arm polyethylene glycol (PEG10K/20K)–thiol as crosslinkers. The six resulting *dsc*HA hydrogels demonstrated solid-like behavior with distinct physical and rheological properties. SEM analysis revealed a decrease in porosity with higher crosslinker MW and maleimide substitution. The swelling ratios of the six hydrogels reached equilibrium at approximately 1 h and ranged from 20% to 35%, indicating relatively low swelling. Degradation rates decreased with increasing maleimide substitution, while crosslinker MW had little effect. Higher maleimide substitution also required greater injection force. Elastic modulus (G′) in the linear viscoelastic region increased with maleimide substitution and crosslinker MW, indicating enhanced firmness. All hydrogels displayed similar creep-recovery behavior, showing instantaneous deformation under constant stress. Alternate-step strain tests indicated that all six *dsc*HA hydrogels could maintain elasticity, allowing them to integrate with the surrounding tissue via viscous deformation caused by the stress exerted by changes in facial expression. Ultimately, the connection between the clinical performance of the obtained *dsc*HA hydrogels used as dermal filler and their physicochemical and rheological properties was discussed to aid clinicians in the selection of the most appropriate hydrogel for facial rejuvenation. While these findings are promising, further studies are required to assess irritation, toxicity, and in vivo degradation before clinical use. Overall, it was concluded that all six *dsc*HA hydrogels show promise as dermal fillers for various facial regions.

## 1. Introduction

Skin tissue is composed of several extracellular matrix (ECM) proteins, including hyaluronic acid (HA), collagen, elastin, and fibrillin [1]. Among these, HA, a polysaccharide, plays a crucial role in maintaining structural integrity and hydration of the skin [2]. The degradation of HA is a main contributor to skin aging, which has led to the development of HA filler products to correct age-related facial contour abnormalities and wrinkles. HA is capable of functioning as a space-occupying device or as a skin booster, significantly enhancing skin quality by stimulating the production of various ECM components [3,4,5]. However, the rheological properties of native HA are viscous-dominant, with the viscous modulus (G″) being greater than the elastic modulus (G′) [6]. This intrinsic rheological characteristic leads to its limitation in its use as a dermal filler for aesthetic rejuvenation. To conquer this problem, HA can be chemically modified through a process called crosslinking, which enhances its viscoelastic properties [7,8]. Crosslinked HA hydrogels exhibit a storage modulus (G′) significantly higher than their loss modulus (G″) [9], thereby increasing the persistence of the filler for soft tissue augmentation and improving its performance in wrinkle reduction and volumization in plastic surgery applications [10].

The association between the clinical performance of crosslinked HA dermal fillers and their physicochemical and rheological properties has been extensively reviewed, which helps clinicians select the most appropriate products for facial rejuvenation [11,12,13]. These links can be summarized as follows: Crosslinked HA hydrogels designated as “stiffer” generally exhibits higher G′ values, lower swelling ratios, and greater cohesivity. Consequently, they are more resistant to enzymatic degradation. Such fillers are well suited for areas where bony structures need replication, as they can withstand the high shear forces found beneath muscles [14]. Nonetheless, excessive cohesion can be disadvantage, as it may limit the hydrogel’s ability to spread evenly, leading to a “bunching up” effect during facial movements and resulting in uneven contouring. On the other hand, “softer” crosslinked HA hydrogels, characterized by lower G′ values, generally have lower HA concentrations and/or degrees of crosslinking. This results in a looser hydrogel network with increased free HA content, making the material more susceptible to enzymatic degradation and quicker elimination from the body [15]. These softer fillers are particularly suitable for finer corrections and provide a more natural feel, making them ideal for treating delicate areas such as tear troughs, lips, and the periorbital region, as well as less dynamic wrinkles. However, their lower crosslinking density typically results in a shorter residence time within the tissue [16].

Currently, 1,4-butanediol diglycidyl ether (BDDE) crosslinking is the most commonly used method for producing crosslinked HA hydrogels as dermal fillers, and in vivo toxicity studies have shown that BDDE has low or no toxicity in rats [17,18]. However, some researchers have raised concerns that chemical crosslinkers may result in cytotoxicity through reactive oxygen species and inflammatory responses, which may decrease the biocompatibility of crosslinked HA [19]. Although reducing the concentration of crosslinkers could improve biocompatibility, it might also reduce therapeutic effectiveness. Recently, click chemistry for gel formation has emerged as a promising strategy for developing injectable hydrogels as dermal fillers, due to its rapid gelation time; high chemoselectivity; mild reaction conditions; and, most importantly, the absence of chemical additives, cytotoxic crosslinking agents, and byproducts during the gel formation process [20,21,22,23]. Among various click reactions, the thiol–maleimide reaction stands out for its ability to specifically functionalize carbohydrates, such as HA and alginic acid [24]. Chemical modifications of HA using its existing hydroxyl (OH) and carboxyl (COOH) groups with maleimide (Mal) and methacrylate (Mac) can facilitate direct grafting onto HA (HA-Mal and HA-Mac). These modifications can then be used for crosslinking with thiol-containing crosslinkers, including dithiothreitol (DTT), four-arm polyethylene glycol (PEG)–thiol (5000 g/mol), and multiarm polyamidoamine (PAMAM) dendrimer crosslinkers, via a thiol–maleimide click reaction [25,26]. Furthermore, a mixed solution of HA-Mal and thiol-containing PEG crosslinkers can be rapidly crosslinked under mild conditions by a Michael addition reaction between thiol groups (-SH) and double bonds (maleimide) to form a single crosslinked HA-based hydrogel. Additionally, free sulfhydryl groups can form disulfide bonds through self-crosslinking to create double self-crosslinked HA-based (*dsc*HA) hydrogels [21,27]. These thiol-crosslinked HA-based hydrogels have shown potential in various biomedical applications, including protein delivery systems, wound healing, and tissue engineering [28].

In this study, we developed double self-crosslinked hyaluronic acid-based hydrogels (*dsc*HA) as a novel and biocompatible alternative to commercial dermal fillers that use BDDE as a crosslinker. Maleimide-substituted hyaluronic acid (HA-Mal) with three different degrees of substitution was synthesized and characterized by 1H NMR and FTIR. Subsequently, double self-crosslinked HA-based hydrogels (*dsc*HA) were fabricated using four-arm PEG-thiol (10,000 g/mol, PEG10K-4SH) and four-arm PEG-thiol (20,000 g/mol, PEG20K-4SH) as thiol-containing crosslinkers. These hydrogels were evaluated for their feasibility as dermal fillers. The effects that the extent of substitution and the molecular weight (MW) of PEG10K-4SH or PEG20K-4SH have on gelation, morphology, injectability, swelling ratio, degradation rate, and rheological properties were characterized. Ultimately, an association between the clinical performance of the obtained *dsc*HA dermal fillers and their physical and rheological properties could be established, thereby assisting clinicians in selecting the most suitable products for facial rejuvenation.

## 2. Results

### 2.1. Synthesis of HA-Mal Conjugates

**Figure 1:**
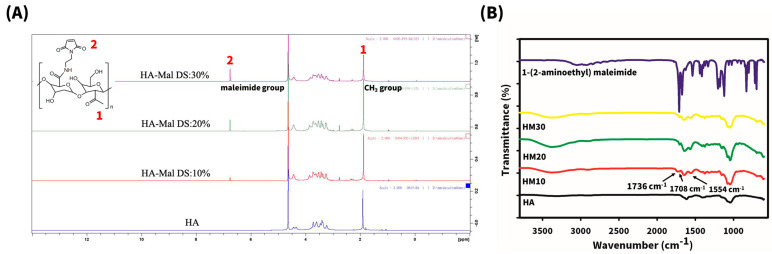
1H NMR (**A**) and FTIR spectra (**B**) of HA and HA-Mal with three different degrees of substitution of maleimide on HA (HM10, HM20, and HM30).

The synthesis of HA-Mal conjugates was first confirmed through 1H NMR spectroscopy. As shown in Figure 1A, new peaks at 6.8 ppm appeared in the spectra of HA-Mal conjugates after the reaction with 1-(2-aminoethyl) maleimide. These peaks increased in intensity with more 1-(2-aminoethyl) maleimide added, consistent with previous reports [29]. Using the peak area at 1.89 ppm corresponding to the methyl group of HA, the degree of maleimide substitution was calculated to be 10%, 20%, and 30%, and these were designated as HM10, HM20, and HM30, respectively.

Further confirmation of the conjugation was provided by FTIR spectroscopy. As shown in Figure 1B, OH-stretching vibration peaks were observed between 3100 and 3690 cm^−1^ in both HA and HA-Mal conjugates. Compared to HA, HA-Mal conjugates exhibited sharp absorption peaks at 1736 cm^−1^, 1708 cm^−1^, and 1554 cm^−1^, corresponding to the stretching vibrations of the newly formed amide bonds between maleimide and HA. These findings demonstrate the successful grafting of the maleimide functional group onto HA.

### 2.2. Preparation of dscHA Hydrogels

**Figure 2:**
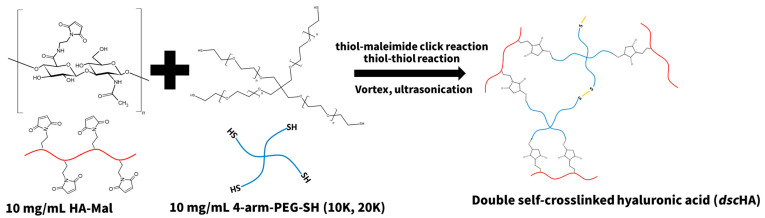
Reaction scheme illustrating the formation of *dsc*HA hydrogels.

The *dsc*HA hydrogels were prepared by mixing the HA with various maleimide substitution degrees (HM10, HM20, and HM30) with four-arm PEG-SH of two different MWs (PEG10K-4SH and PEG20K-4SH), both at the same concentration of 10 mg/mL, as shown in Figure 2. The detailed formulations are described in Table 1 and are designated as HM10-4SH10K, HM10-4SH20K, HM20-4SH10K, HM20-4SH20K, HM30-4SH10K, and HM30-4SH20K.

The activities of double bonds in various molecular structures significantly influenced the reaction efficiency of the thiol–Michael addition click reaction. Previous reports demonstrated that the reaction efficiency of propylmaleimide was higher and faster than those of other -enes, including ethyl methacrylate, diethyl maleate, and dimethylacrylamide [30]. Therefore, it was anticipated that the formation of *dsc*HA hydrogels would involve two main covalent reactions: the formation of a stable primary network via a highly efficient thiol–maleimide click reaction, which provides robust structural integrity, and the establishment of a reductively degradable secondary network through oxidation-induced self-crosslinking among thiol groups, resulting in disulfide linkages that contribute to the hydrogel’s degradability under reductive conditions.

### 2.3. Physical Characterization of dscHA Hydrogels

#### 2.3.1. Morphology

**Figure 3:**
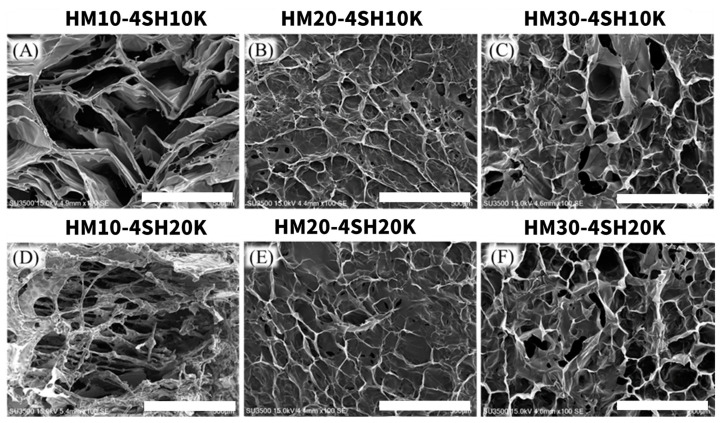
SEM images of six *dsc*HA hydrogels (HM10-4SH10K (**A**), HM20-4SH10K (**B**), HM30-4SH10K (**C**), HM10-4SH20K (**D**), HM20-4SH20K (**E**), and HM30-4SH20K (**F**). (Scale bar: 500 µm).

SEM images were used to evaluate the three-dimensional network structure of lyophilized *dsc*HA hydrogels, as shown in Figure 3. The porosity observed in all *dsc*HA hydrogels appeared to decrease as both the MW of the thiol-containing crosslinker and the degree of maleimide substitution on the HA backbone increased. This phenomenon can be attributed to the increased crosslinking density resulting from higher degrees of maleimide substitution and longer PEG chains. Reduced porosity often correlates with a denser and more uniform hydrogel network structure, as previously observed in crosslinked hydrogels with high crosslinking densities [31]. The reduced porosity across all six *dsc*HA hydrogels also indicated a more homogeneous distribution of crosslinked HA chains within the hydrogel network, which is likely due to the greater cohesion among the crosslinked HA chains, leading to higher cohesivity in all six *dsc*HA hydrogels [32].

#### 2.3.2. Swelling Ratio and Degradation Behavior of *dsc*HA Hydrogels

**Figure 4:**
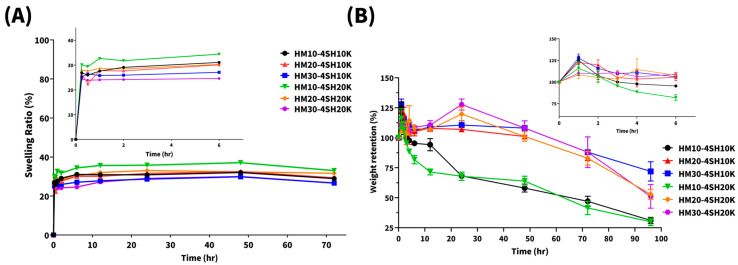
The swelling ratio profiles (**A**) and degradation profiles (**B**) for HAs with various levels of maleimide substitution and thiol-containing crosslinkers with two different MWs (designated as HM10-4SH10K, HM10-4SH20K, HM20-4SH10K, HM20-4SH20K, HM30-4SH10K, and HM30-4SH20K, respectively).

The swelling ratio profiles of all six *dsc*HA hydrogels, shown in Figure 4A, fluctuated between 26% and 33% and reached equilibrium after approximately 1 h. The *dsc*HA hydrogel with the most maleimide substitution (30%) and the larger MW of the thiol-containing crosslinker (20 K), HM30-4SH20K, exhibited the lowest swelling ratio (26%). In comparison, the *dsc*HA hydrogel with the least maleimide substitution (10%) and the same MW of thiol-containing crosslinker (20 K), HM10-4SH20K, showed the highest swelling ratio (33%). Notably, the swelling ratios of various conventional BDDE-crosslinked HA are typically over 50% [33,34]. In comparison, all *dsc*HA hydrogels exhibited lower swelling ratios.

For a more in-depth comparison after the first hour, it was found that for *dsc*HA hydrogels with 30% maleimide substitution, increasing the MW of the thiol-containing crosslinker (4SH20K) resulted in a lower swelling ratio compared to those with a lower-MW crosslinker (PEG10K-4SH). Conversely, for hydrogels with 10% maleimide substitution, increasing the MW of the crosslinker resulted in a higher swelling ratio compared to those with PEG10K-4SH. For the intermediate level of maleimide substitution (20%), the swelling ratio slightly increased with the higher MW of PEG20K-4SH compared to PEG10K-4SH. These findings were consistent with previous studies, which demonstrated that increasing the MW of the crosslinker can result in a loosely crosslinked network structure, thereby increasing the swelling ratio in systems [35].

Maleimide is thought to be a hydrophobic moiety that results in the decrease of hydrophilic–lipophilic balance (HLB) in HA when -COOH groups are substituted with -Mal group, causing more hydrophobicity with a higher degree of substitution. Therefore, the absorption of water into *dsc*HA hydrogel should gradually decline with an increasing degree of maleimide substitution (HM10 > HM20 > HM30). On the other hand, four-arm PEG-thiol is recognized as a hydrophilic moiety, and its hydrophilicity is predicted to increase with increasing MW of the PEG chain (4SH20K > 4SH10K). The higher crosslinking density in hydrogels with 30% maleimide substitution and PEG20K-4SH likely results in fewer free PEG arms, leading to increased hydrophobicity in HM30-4SH20K compared to HM30-4SH10K. In contrast, hydrogels with 10% maleimide substitution and PEG20K-4SH have a lower crosslinking density, leading to more free PEG arms and thus higher hydrophilicity in HM10-4SH20K compared to HM10-4SH10K. Consequently, the swelling ratio followed the order HM30-4SH20K < HM30-4SH10K < HM20-4SH20K ≈ HM20-4SH10K ≈ HM10-4SH10K < HM10-4SH20K. Nonetheless, all six *dsc*HA hydrogels exhibited a relatively low swelling ratio, ranging between 26% and 33%.

The swelling ability of the *dsc*HA hydrogel depends mainly on the structure of its polymer network (the crosslinking density) and secondarily on the hydrophilic nature of crosslinker. A more tightly crosslinked hydrogel network (higher crosslinking density) limits the ability of the hydrogel to expand during water uptake compared to a looser network (lower crosslinking density) [36]. The extent of crosslinking also affects the polymer chains’ ability to remain bonded, reducing the spacing between chains and limiting the penetration of water molecules. As a result, highly crosslinked hydrogels used as dermal fillers have lower fluid uptake due to their denser network, leading to a reduced ability to expand [37]. Dermal fillers with lower swelling ratios exhibit reduced water molecule binding capacity, leading to less swelling compared to those with higher swelling ratios [38]. A previous study also illustrated the association between swelling ratio and the hydrogel’s hydro-action, reflecting its capacity to expand post-injection by binding with water molecules [39]. While hydrogel expansion after injection is desirable in certain clinical treatments, excessive swelling can lead to undesired outcomes such as palpability and edema. Such challenges highlight the importance of optimizing swelling behavior for aesthetic treatments.

#### 2.3.3. Degradation Behavior of *dsc*HA Hydrogels

Hyaluronidase is an intrinsic enzyme that catalyzes the degradation of HA by hydrolyzing the bond between N-acetylglucosamine and glucuronic acid [15]. The structural difference between natural HA and crosslinked HA alters enzymatic degradation by hyaluronidases, resulting in a longer residence time at the injection site, which enhances the product’s efficacy. The degradation profiles of the six *dsc*HA hydrogels observed in phosphate-buffered saline (PBS) buffer with the addition of hyaluronidase are plotted in Figure 4B. In the first hour, the profiles were similar to those observed in the swelling study. Then, *dsc*HA hydrogels degraded in a time-dependent manner under the effect of hyaluronidase.

The results showed that the degradation rate decreased as the degree of maleimide substitution increased (HM10 > HM20 > HM30). However, the MW of the thiol-containing crosslinker had only a slight effect on the degradation rate. Specifically, weight retention after 96 h was approximately 70% for both HM30-4SH10K and HM30-4SH20K, ~50% for both HM20-4SH10K and HM20-4SH20K, and ~25% for both HM10-4SH10K and HM10-4SH20K. Compared with conventional BDDE-crosslinked HA hydrogels, which are rapidly and completely degraded by hyaluronidase within 24 h [33,40], all six *dsc*HA hydrogels exhibited a significantly slower degradation rate. The slower degradation can be attributed to the increased crosslinking density resulting from the double crosslinking mechanism, which creates a barrier that hinders hyaluronidase from accessing its binding sites within the hydrogel. Additionally, the hydrophobic nature of the maleimide substitution made the HA chains less soluble, further reducing their exposure to hyaluronidase and slowing down enzymatic degradation [41].

#### 2.3.4. Injection Force of *dsc*HA Hydrogels

The injectability of hydrogels used as dermal filler is associated with the quality, safety, and efficacy outcomes of specific medical aesthetic interventions [42]. In addition, the clinical outcome of dermal filler applications depends on the experience and expertise of the clinician, with precise control over the injection process being important to ensure that the correct amount of hydrogel is delivered to the appropriate anatomical site and depth [43,44,45]. Therefore, the injectability of dermal filler products has been a key consideration for development of *dsc*HA hydrogels as dermal fillers.

**Figure 5:**
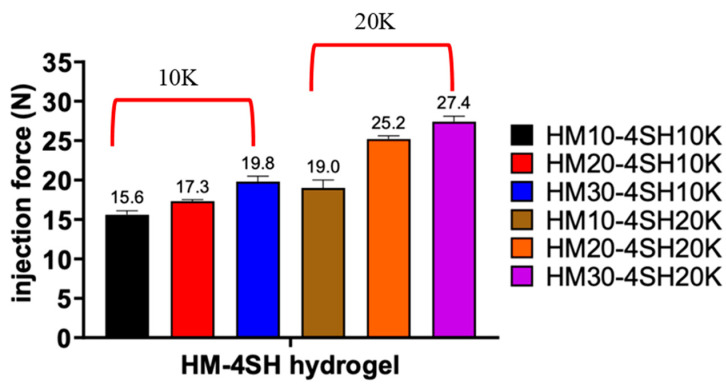
Injection force through a 26 G needle measured for six *dsc*HA hydrogels (HM10-4SH10K, HM10-4SH20K, HM20-4SH10K, HM20-4SH20K, HM30-4SH10K, and HM30-4SH20K).

In this study, the injection force was measured for six kinds of *dsc*HA hydrogels, and the results are illustrated in Figure 5. It appeared that increasing maleimide substitution resulted in more injection force required to push hydrogel out of the injection needle. Similarly, increasing MW of the thiol-containing crosslinker also led to an increase in injection force, but the effect was less pronounced than that of increasing maleimide substitution. These findings also suggest that more stable hydrogel structures require more injection force.

### 2.4. Rheology Study

Understanding the rheological properties of *dsc*HA hydrogels as dermal fillers is essential for clinicians in selecting the most suitable product for specific indications, facial regions, and anatomical layers [46]. *dsc*HA hydrogels with varying rheological characteristics must respond appropriately to the applied forces to achieve optimal defect correction.

Five primary rheological parameters are typically used to describe the viscoelastic properties of *dsc*HA hydrogels, including the storage modulus (G′), the loss modulus (G″), the complex modulus (G*), tangent delta (tan δ = G″/G′), and the complex viscosity (η*). These parameters provide a comprehensive assessment of the hydrogel’s viscoelastic behavior. Therefore, when evaluating the rheological properties of *dsc*HA hydrogels intended for use as dermal fillers, these measurements should be considered as key indicators of performance [47].

#### 2.4.1. Oscillatory Amplitude and Frequency Sweep (G′ and G″)

**Figure 6:**
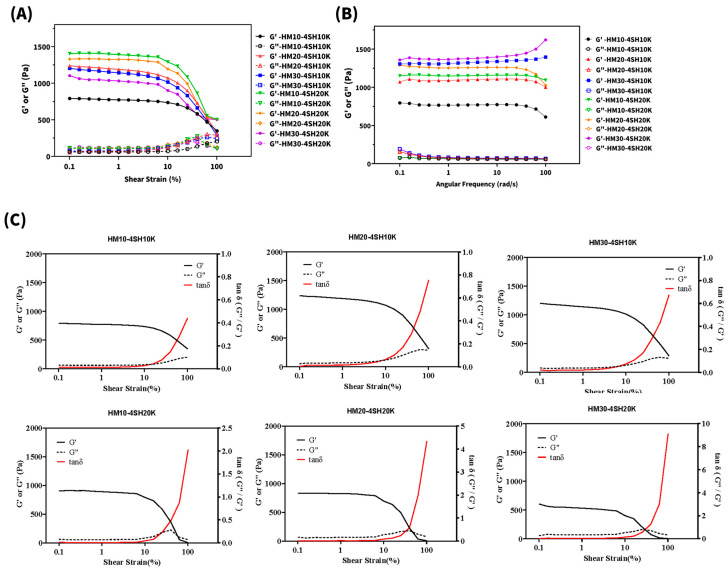
Rheological evaluation of *dsc*HA hydrogels. Amplitude sweep (**A**) and frequency sweep (**B**) of the six *dsc*HA hydrogels, showing the linear viscoelastic (LVE) region and gel behavior. Tan δ values (**C**) of the six *dsc*HA hydrogels, indicating whether the behavior is elastic-dominant or viscous-dominant.

The oscillatory shear stress test is used to measure the storage modulus (G′) and the loss modulus (G″). This test must be performed in oscillation mode within the LVE region. To determine the LVE region for all six *dsc*HA hydrogels, an oscillatory amplitude sweep was first conducted at a strain range of 0.1% to 100%; the results are shown in Figure 6A. The LVE region defines the range in which the hydrogel network structure remains intact during testing. In this region, both G′ and G″ exhibit constant, plateau-like values. The limiting value of the LVE region, also referred to as the linearity limit, serves as a reference for selecting the optimal strain for frequency sweep tests.

The plots of both G′ and G″ versus strain illustrated in Figure 6A demonstrate that all six curves of the G′ plots maintain a plateau before the LVE regions (at approximately 10% shear strain), after which a continuous decline is observed, indicating a gradual breakdown of the hydrogel superstructure. This gradual breakdown explains the “creamy” behavior of the hydrogels during structural deformation, instead of brittle fracturing behavior (or non-creamy behavior), which should manifest as a sharp downturn at a strain beyond the linearity limit of the LVE region. Creamy behavior for all six *dsc*HA hydrogels after a gradual breakdown of the superstructure might also indicate that higher cohesivity could be expected for all six *dsc*HA hydrogels.

The oscillatory amplitude sweep results further showed that the G′ curves for all six hydrogels exhibited a smooth downturn around 10% strain, whereas the G″ curves displayed a slight gradual increase with increasing strain. However, no crossover between G′ and G″ was observed, even at the highest shear strain of 100%, indicating that the hydrogels did not transition from an elastic solid to a viscous liquid but instead deformed while maintaining a creamy consistency. Based on these findings, a 0.5% strain within the LVE region was selected for conducting oscillatory frequency sweeps across a frequency range of 0.1 Hz to 10 Hz to evaluate the gel strength of the six *dsc*HA hydrogels. The weak dependence of G′ on angular frequency confirms the covalent, permanent nature of the crosslinked network.

Figure 6B illustrates oscillatory frequency sweep profiles for the six *dsc*HA hydrogels. For all the hydrogels, G′ remained at a plateau across the 0.1–10 Hz range, indicative of a stable crosslinked network and solid-like behavior. In the comparison of the plateau value of G′ (at 1 Hz) for all six *dsc*HA hydrogels, it was demonstrated that G′ increased with both the degree of maleimide substitution (HM30 > HM20 > HM10) and the MW of the thiol-containing crosslinker (PEG20K-4SH > PEG10K-4SH), resulting in the following order of G′: HM10-4SH10K (576.0 Pa) < HM10-4SH20K (697.3 Pa) < HM20-4SH10K (736.8 Pa) < HM20-4SH20K (1042.0 Pa) < HM30-4SH10K (1332.8 pa) < HM30-4SH20K (1389.4 pa). Although an increase in the degree of maleimide substitution is expected to increase the crosslinking density, the increase in G′ with higher-MW crosslinkers may reflect enhanced chain entanglement and a more robust three-dimensional network structure. At higher frequencies, the two hydrogels with the highest level of maleimide substitution (HM30-4SH10K and HM30-4SH20K) exhibited a gradual increase in G′, indicating further stiffening and more pronounced “solid-like” behavior. By contrast, the other four hydrogels with lower levels of maleimide substitution (HM20 and HM10) demonstrated a gradual decline in G′ at higher frequencies, indicating a transition toward more “liquid-like” behavior as frequency increased.

The ratio of G″ to G′, represented as tan δ (phase angle δ), as shown in Figure 6C, illustrates the relative contributions of the viscous and elastic moduli across different frequencies. A hydrogel with a low tan δ is predominantly elastic, meaning it deforms under shear stress but regains its original shape once the stress is removed. In contrast, a hydrogel with a higher tan δ (indicating greater viscosity, typically observed at lower frequencies or over extended times) deforms and flows more readily. Therefore, the phase angle δ is often associated with a product’s capacity to migrate from the injection site, and hydrogels with a low phase angle can be expected to exhibit limited migration following injection.

Furthermore, the tan δ value can be indicative of the cohesivity of hydrogels, which refers to their ability to deform without breaking and their malleability under shear stress, driven by the molecular affinity within the hydrogel network. Hydrogels with greater tan δ values (between 0.5 and 1) behave similarly to warm butter spread on toast, dissipating energy more readily due to their lower viscosity. This increased deformability allows these hydrogels to glide or flow more easily and withstand higher shear stress without fracturing. In comparison, hydrogels with lower tan δ values (between 0 and 0.5) are more rigid, resembling refrigerated butter on toast, and are more prone to fragmentation under shear stress. Consequently, the cohesivity of a hydrogel plays a crucial role in ensuring its even spread and smooth adaptation to surrounding tissue [48]. On the other hand, a hydrogel with a greater G′ is ideal for deep, targeted deposition, as it resists deformation and minimizes distribution of the hydrogel into surrounding tissue. Hydrogels with high G′ values feel firmer within the tissues and are particularly useful in cases where structural definition, precision, or tissue lifting is needed.

#### 2.4.2. Creep Recovery and Alternate-Step Strain Test

**Figure 7:**
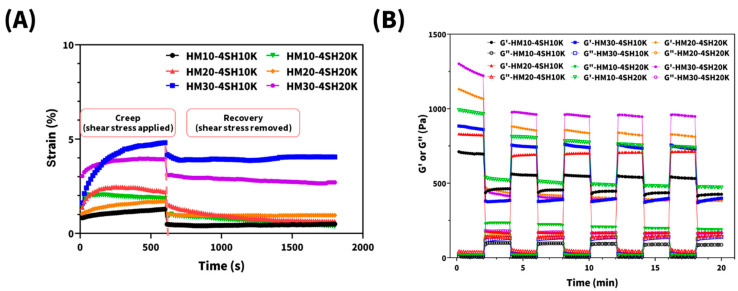
Creep-recovery experiments (constant stress) were performed with an applied shear stress of 5 Pa for 10 min followed by 20 min of recovery (**A**), and alternate-step strain tests with five repetitions of shear-stress application and relaxation (**B**) were performed to study the deformation and recovery of the hydrogel network.

To further evaluate the rheological properties and investigate the architectures of the hydrogels, creep-recovery experiments were conducted. As shown in Figure 7A, the creep-recovery curves exhibited similar shapes across all six *dsc*HA hydrogels. Upon the application of constant stress, all hydrogels exhibited an instantaneous deformation, followed by a steady increase in strain over time, displaying varying degrees of time-dependent behavior. A noticeable difference was observed when the stress was removed: hydrogels with a stronger time-dependent trend, such as HM20-4SH10K and HM30-4SH10K, showed delayed recovery, while others recovered more quickly and completely. These results suggest that structural rearrangements within the hydrogels display delayed recovery, likely due to the presence of free and dangling polymer chains.

Furthermore, the extent of strain at the end of the loading phase decreases as the degree of maleimide substitution increases. After the removal of shear stress, most of the *dsc*HA hydrogels showed an instantaneous recovery to a plateau, followed by a more pronounced permanent strain, indicating the presence of predominant viscous deformation and partly viscoelastic, fluid-like behavior. Regardless of the degree of maleimide crosslinking, the slippage of dangling chains in the hydrogel network prevented full recovery to their initial state, resulting in permanent deformation after the unloading phase.

The alternate-step strain test was performed to study the deformation and recovery of the hydrogel network under repeated stress. The results of five repeated cycles of shear-stress application and subsequent relaxation are illustrated in Figure 7B. At low strain (1%), all six hydrogels exhibited higher G′ than G″ values, indicating a stable hydrogel network. When subjected to higher strain (100%), G′ decreased and G″ increased, though G′ remained higher than G″, indicating that the hydrogels maintained their viscous gel state. Upon returning the hydrogels to 1% strain after the first cycle, G′ and G″ did not return to their original values but returned to the same order as in the initial state. This indicates that all six *dsc*HA hydrogels experienced permanent viscous deformation due to the slippage of dangling chains within the network. However, after three additional repetitions of the stress–relaxation cycle, the values of G′ and G″ remained consistent with those observed in the second cycle, suggesting that a rigid gel was reformed after repeated cycles of stress and relaxation. These findings demonstrate that all six *dsc*HA hydrogels retained a degree of elasticity, allowing them to be molded and integrate with surrounding tissue through viscous deformation. This behavior is crucial for dermal fillers, as it enables the hydrogels to accommodate the dynamic forces exerted during facial expressions.

## 3. Discussion

In this study, *dsc*HA hydrogels composed of maleimide-substituted HA (with three different degrees of maleimide substitution: HM10, HM20, and HM30) and thiol-containing crosslinkers (four-arm PEG-SH with two different MWs: PEG10K4SH and PEG20K4SH), were successfully synthesized. The primary network was formed through a thiol/maleimide click reaction, while a secondary network was created by oxidation-induced disulfide bond formation among thiol groups. The formation of viscous, fluid-like hydrogel in the absence of maleimide substitution indicates that the gel strength of the *dsc*HA hydrogels was primarily due to covalent linkages formed by the thiol–maleimide click reaction. Notably, increasing the degree of maleimide substitution increased the gel strength (G′). Even at low maleimide substitution (10%) and an HA concentration of 10 mg/mL, the gel strength of the *dsc*HA hydrogel was higher than that of most commercially available BDDE-crosslinked products (typically at 20 mg/mL), which generally exhibit gel strengths below 500 Pa [49]. This result demonstrated the high efficiency and superior strength of the thiol–maleimide crosslinking reaction. Additionally, the use of four-arm PEG-SH as a three-dimensional crosslinker contributed to the formation of a three-dimensional spatial structure, further enhancing the gel strength of *dsc*HA hydrogels compared to HA hydrogels crosslinked with BDDE, the most commercially available crosslinked HA.

Crosslinked HA-based hydrogels as dermal fillers play a role as an important product for minimally invasive aesthetic procedures, with a wide range of products now available for clinicians. Several reviews revealed that the science-based evaluation of crosslinked HA-based hydrogels, particularly their rheological properties, is a valuable tool for guiding clinicians in product selection, injection techniques, and optimal injection depths [10,11,12,43,50]. Salvatore Piero Fundarò et al. identified four key clinical stages in the application of HA-based dermal fillers: injection, tissue integration, volume restoration, and hydration [50]. In each of these phases, specific rheological and biophysical characteristics of *dsc*HA hydrogels influence their behavior, from injection to their long-term presence in soft tissues.

The injection stage includes both the passage of the filler through the needle and its immediate integration into soft tissues. Injectability is primarily determined by the yield stress and dynamic viscosity under steady-state conditions, which influence the smooth extrusion of the filler through fine needles. Most dermal filler products are delivered via 30 G or 27 G needles, while some volumizing fillers may require 25 G or 23 G needles. It is crucial to balance sufficient elasticity (G′ or G*) for volume restoration with low viscosity (G″ or η*) to ensure smooth extrusion through fine needles. As shown in Table 2, the six *dsc*HA hydrogels in this study exhibited G′ (G*) values (Pa at 1 Hz) ranging from 576.04 (576.06) to 1389.4 (1389.7) and η* (G″) values (Pa·s/Pa at 1 Hz) ranging from 91.30 (4.580) to 220.0 (29.11), offering a broad selection of hydrogels suitable for different clinical needs, ensuring ease of injection with minimal pressure on the syringe plunger.

In the tissue integration step, once extruded through the needle, the *dsc*HA hydrogels enter and spread into the soft tissue, resulting in the initial integration of the *dsc*HA hydrogels with surrounding tissue. Successful integration is crucial for the final correction of defects and for avoiding nodules caused by material accumulation. Hydrogels with lower η* values contribute to more homogeneous spreading, leading to a softer feel and reduced palpability [51]. Therefore, viscosity is a rheological feature that can be used to identify the correct injection plane of *dsc*HA hydrogel in the dermis. Additionally, tan δ, the ratio of G″ to G′, provides insight into a hydrogel’s spreadability. Hydrogels with a higher tan δ (closer to one) are more viscous and spread easily within the dermal collagen network, whereas those with lower tan δ values are more elastic and are better suited for lifting or volume restoration [52]. Therefore, tan δ might be recognized as a parameter that reveals the liquid-gel classification of *dsc*HA hydrogels [53,54]. As showed in Table 2, all six *dsc*HA hydrogels had tan δ values far below 1.0 (ranging from 0.008 to 0.021), indicating that they are classified as “elastic gels”. These gels are suitable for deep injections where high lifting or volume restoration is required, rather than for superficial injections or fine-line corrections.

The primary function of volumizing *dsc*HA used as a dermal filler is to restore soft tissue volume and resist deformation after injection. The elastic modulus (G′) is a key determinant of a hydrogel’s ability to lift tissue and resist deformation, as it indicates the filler’s resistance to shear forces generated by gravity and facial muscle movement. Typically, hydrogels with higher G′ values offer greater lifting capacity and are best injected deep into soft tissues (e.g., deep-fat compartments or the preperiosteal plane), where they are neither palpable nor visible [37,55,56]. As shown in Table 2, the G′ values for the six *dsc*HA hydrogels in this study ranged from 576.04 Pa to 1389.4 Pa, allowing clinicians to select the most appropriate filler for each injection site based on the required lifting capacity.

However, after their injection, HA dermal fillers are subjected to shear stress, vertical compression, and stretching forces, leading to deformation. To achieve successful volume restoration in deep-fat compartments, the filler must remain compact and resist fragmentation under vertical pressure. Cohesivity, defined as the internal adhesion forces between individual crosslinked HA units, is crucial for preventing fragmentation when the filler is subjected to external forces [57]. Hydrogels with high cohesivity resist compression, maintaining their original shape after injection. In this study, all six *dsc*HA hydrogels demonstrated high G′ values and cohesivity, confirming their suitability for volume restoration in facial tissues.

Following injection, HA fillers attract water, restoring tissue hydration and contributing to volume augmentation. The swelling ratio indicates the hydrogel’s water uptake capacity; hydrogels with a higher swelling ratio provide greater volume enhancement due to water binding. However, hydrogels with higher G′ values have lower swelling ratios because the dense crosslinking prevents water penetration. As shown in Figure 4A, the swelling ratios of the six *dsc*HA hydrogels decreased with increasing G′, ranging from 20% to 35%. This lower swelling ratio indicates that these fillers are not ideal for areas prone to edema, such as the tear trough or lips, which require soft fillers with low G′ and low swelling ratios.

Finally, it is important to note that the only potential component in *dsc*HA hydrogels that could raise cytotoxicity concerns is four-arm PEG-SH. PEG, a synthetic polymer composed of a repeated ethylene glycol subunit, is widely recognized as non-antigenic, non-immunogenic, and non-toxic. It has been approved by the U.S. Food and Drug Administration (FDA) for use in dermal, oral, and intravenous pharmaceutical applications [58]. Cellular uptake studies of PEG polymers with varying MWs (e.g., PEG600, PEG2K, PEG4K, and PEG10K) in MCF-7 cells have confirmed that the cytotoxicity of PEG is minimal. As the MW of PEG increases, its ability to penetrate cell membranes decreases, consistent with its low oral bioavailability. Thus, in vivo animal studies to assess the cytotoxicity of *dsc*HA hydrogels were deemed unnecessary in this preliminary rheological characterization study.

## 4. Conclusions

It was concluded that six *dsc*HA hydrogels, composed of HA with three different degrees of maleimide substitution (HM10, HM20, and HM-30) crosslinked with four-arm PEG-SH of two different MWs (PEG10K4SH and PEG20K4SH), were successfully prepared. These six *dsc*HA hydrogels, if used as dermal fillers, could provide solid-like behavior with various extents of elasticity for projection and volumization. Furthermore, they demonstrated moldability, which allows them to integrate smoothly with the surrounding tissue through viscous deformation, potentially caused by the stresses exerted during facial expressions. Overall, the six *dsc*HA hydrogels show significant promise as dermal fillers for various facial regions. A clinical study to evaluate their efficacy and safety for the correction of facial defects is under way.

## 5. Materials and Methods

### 5.1. Materials

Sodium hyaluronate (HA, ~100 kDa weight-average MW) was purchased from TOP RHYME INTERNATIONAL Co., Ltd. (New Taipei City, Taiwan). N-Hydroxysuccinimide (NHS), 2-morpholinoethanesulfonic acid (MES) hydrate, 1-ethyl-3-(3-dimethylaminopropyl) carbodiimide (EDC) hydrochloride salt, 1-(2-aminoethyl) maleimide hydrochloride salt, and 1-(2-aminoethyl) maleimide trifluoroacetate salt were purchased from Sigma Aldrich (St. Louis, MO, USA). Four-arm PEG-SH crosslinkers (MW 10K and 20K g/mol) were obtained from biochempeg (Watertown, MA, USA). All reagents used were of analytical grade.

### 5.2. Synthesis of HA-Mal Conjugates

HA-Mal conjugates were synthesized following a procedure similar to the one described in a previous study with a minor modification [21]. Initially, 1 mmol of HA (100 kDa) was dissolved in a 0.2 M MES buffer solution (100 mL) at a temperature of 25 °C and a pH of 5.0, and the mixture was stirred until complete dissolution. Subsequently, 0.3–3 mmol of maleimide (Mal), 2 mmol of NHS, and 3 mmol of EDC were added to the HA solution, and the pH was adjusted to 5.5 using 1 M NaOH solution. The reaction was allowed to proceed for 24 h with continuous stirring at 25 °C. Following the reaction, the resulting mixture was subjected to dialysis using a dialysis tube with an MW cutoff (MWCO) of 6–8 kDa. The dialysis was carried out using acidic deionized water (pH = 3.5) as the dialysis medium for a duration of 3 days. After dialysis, the solution was lyophilized at −20 °C under vacuum for 3 days to obtain a spongy solid product of HA-Mal. The degree of substitution of maleimide on HA was determined by ^1^H NMR analysis using an Agilent 600 MHz DD2 NMR instrument (Santa Clara, CA, USA) at 25 °C, with D2O as the solvent. Fourier-transform infrared spectroscopy (FTIR) was performed by mixing HA or HA-Mal with KBr and analyzing the samples using a Bruker Tensor 27 FT-IR spectrometer (Billerica, MA, USA) in the range of 500–4000 cm^−1^ with a resolution of 4 cm^−1^.

### 5.3. Preparation of dscHA Hydrogels

To prepare *dsc*HA, 40 mg of HA and HA-Mal with three different degrees of substitution (designated as HM10 (10%), HM20 (20%), and HM30 (30%)) were separately weighed into a 5 mL Eppendorf tube. A volume of 2 mL of 0.01 M PBS (pH 5.0) was added to this tube, and the mixture was subjected to repeated vortexing (20 s each time) and sonication (30 min each time) until complete dissolution. Into another 5 mL Eppendorf tube, 40 mg of four-arm PEG10K-SH or four-arm PEG20K-SH was separately weighed. A volume of 2 mL of 0.01 M PBS (pH 5.0) was added to the tube, and the mixture was similarly treated, being subjected to repeated vortexing (20 s/each time) and sonication (30 min/each time) until complete dissolution. After completely dissolving, equal amounts (0.5 mL) of HA and HM10, HM20, or HM30 were placed in a 24-well culture plate, and 0.5 mL each of four-arm PEG10K-SH and four-arm PEG20K-SH PBS solution were added into each 24-well culture plate to make a final volume of 1 mL. Next, the pH value of the resultant hydrogel solution was adjusted to 7.0 with 1 M NaOH solution to activate the thiol–maleimide click reaction, forming the thiol-ene linkage. Finally, the culture plate was placed in a shaker oven at 37 °C for 24 h to facilitate the formation of thiol–thiol (S-S) linkages among the thiol-containing crosslinker (four-arm PEG-SH). The detailed formulation of eight dermal filler hydrogels are listed in Table 1. Accordingly, eight dermal filler hydrogels composed of various extents of maleimide substitution on HA (HM0, HM10, HM20, and HM30) and thiol-containing crosslinkers with various PEG chain MWs (4SH10K and 4SH20K) were designated HM0-4SH10K, HM0-4SH20K, HM10-4SH10K, HM10-4SH20K, HM20-4SH10K, HM20-4SH20K, HM30-4SH10K, and HM30-4SH20K, respectively.

### 5.4. Physical and Rheological Characterization of dscHA Hydrogels

For evaluation of the morphology and microstructure of *dsc*HA hydrogels, the hydrogels were frozen at −80 °C and subsequently lyophilized for 3 days. The lyophilized hydrogels were observed using scanning electron microscopy (SEM, Hitachi SU3500, Tokyo, Japan). Rheological measurements were performed on an Anton Paar MCR302 Rheometer (Anton Paar GmbH, Graz, Austria) with a 25 mm parallel plate and 1.0 mm gap. The storage modulus (G′) and loss modulus (G″) were measured. Strain amplitude sweep experiments were performed on samples from 0.1% to 100% to determine the linear viscoelastic region (LVR). Approximately 1.0 mL of *dsc*HA hydrogel was applied to the plate, and the temperature equilibrium was set at 25 °C before starting the strain amplitude sweep measurement. Frequency sweep experiments were conducted over a frequency range of 0.1–100 rad/s, with a strain of 0.5% at 25 °C. Creep experiments were performed with a shear stress of 5 Pa for 10 min, followed by 20 min of recovery. The self-recovering behavior was assessed using alternate-step strain test measurements, in which small alternate-step strains of 1% for 120 s and large strains of 100% for 120 s were applied and repeated five times with the frequency set at 10 rad/s. For the swelling ratio, *dsc*HA hydrogels were initially weighed (W0), immersed in 50 mL of 0.01 M PBS buffer (pH = 7.4), and then placed in a constant-temperature bath at 37 °C. The hydrogels were weighed (W1) at a specific time interval until equilibrium swelling was achieved. The swelling ratio of the hydrogel was calculated using the following formula: swelling ratio (%) = 100 (W1 − W0)/W0. For degradation studies, *dsc*HA hydrogels were initially weighed (W0) and immersed in 50 mL of 0.01 M PBS buffer (pH = 7.4) supplemented with 60 units/mL hyaluronidase at 37 °C. At specific time points, the supernatant was removed, and the remaining weight (Wᵣ) was measured. The weight retention was calculated using the following formula: weight retention (%) = 100 Wr/W0. The injectability of each HA filler was assessed by measuring the force required for injection. Briefly, the *dsc*HA hydrogels were individually filled into a 1 mL syringe with a 26 G needle (Terumo, Tokyo, Japan). The extrusion force was measured using a digital force gauge with a 50 kg load cell. Each experiment was performed in triplicate.

### 5.5. Statistical Analysis

All data are presented as the mean ± standard deviation (SD). A one-way analysis of variance (ANOVA) was used to determine significant differences. *p* < 0.05 (*) was considered to be significant, and *p* < 0.01 (**) was considered particularly significant.

## Figures and Tables

**Table 1:** *dsc*HA hydrogels composed of HA with various degrees of maleimide substitution and thiol-containing crosslinkers with various PEG chain MWs.

*w*/*w* (mg)		Maleimide-Modified HA			
4-arm PEG-SH	MW/DS	HM0	HM10	HM20	HM30
	PEG-10K (4SH10K)	HM0-4SH10K (10/10)	HM10-4SH10K (10/10)	HM20-4SH10K (10/10)	HM30-4SH10K (10/10)
	PEG-20K (4SH20K)	HM0-4SH20K (10/10)	HM0-4SH20K (10/10)	HM0-4SH20K (10/10)	HM0-4SH20K (10/10)

**Table 2:** Viscoelastic properties of HA-based dermal fillers at frequencies of 0.1, 1, and 4 Hz (shear strain = 0.5%, T = 25 °C).

Samples	G′ (Pa)			G″ (Pa)			Tan δ			G* (Pa)			η* (Pas)		
	0.1	1	4	0.1	1	4	0.1	1	4	0.1	1	4	0.1	1	4
HM10-4SH10K	574.36	576.04	596.86	7.857	4.580	4.606	0.014	0.008	0.008	574.41	576.06	596.88	910.38	91.30	23.76
HM20-4SH10K	724.04	736.83	736.87	21.50	14.99	13.13	0.030	0.020	0.018	724.36	736.98	736.98	1148.0	116.8	29.34
HM30-4SH10K	1306.1	1332.8	1352.9	35.89	22.51	21.07	0.027	0.017	0.016	1306.6	1333.0	1352.1	2070.0	211.0	53.80
HM10-4SH20K	690.48	697.29	702.94	9.561	6.194	6.274	0.014	0.009	0.009	690.55	697.32	702.97	1090.0	111.0	27.99
HM20-4SH20K	1014.4	1042.0	1072.6	24.22	20.77	20.56	0.024	0.020	0.019	1014.7	1042.3	1072.8	1610.0	165.0	42.71
HM30-4SH20K	1364.3	1389.4	1422.2	46.51	29.11	28.38	0.034	0.021	0.020	1365.1	1389.7	1422.6	2160.0	220.0	56.63

## Data Availability

The data will be available upon reasonable request.
